# How do nutrition professionals working in low‐income countries perceive and prioritize actions to prevent wasting? A mixed‐methods study

**DOI:** 10.1111/mcn.13035

**Published:** 2020-06-08

**Authors:** Scott B. Ickes, Christina Craig, Rebecca A. Heidkamp

**Affiliations:** ^1^ Department of Applied Health Science Wheaton College Wheaton Illinois USA; ^2^ Departments of Global Health and Health Services University of Washington School of Public Health Seattle Washington USA; ^3^ Independent Consultant Atlanta Georgia USA; ^4^ Department of International Health Johns Hopkins Bloomberg School of Public Health Baltimore Maryland USA

**Keywords:** acute malnutrition, interventions, nutritional status, stunting, wasting prevention

## Abstract

Despite a shared commitment to achieving global nutrition targets, development and emergency‐humanitarian actors tend to prioritize different nutrition outcomes and actions. New approaches are needed to bridge the divide between these communities and to strengthen the overall evidence base for prevention of wasting. To better understand how these different groups perceive and prioritize actions for wasting prevention, key informant interviews (*n* = 21) were conducted, and an online survey was fielded among nutrition professionals working in low‐income countries (*n* = 107). Additionally, nutrition policy and strategy documents for select global and country institutions (*n* = 12) were analysed to identify interventions and approaches for addressing different forms of undernutrition. The findings of this study suggest that at both global and country levels, development actors tend to prioritize stunting prevention, and emergency‐humanitarian actors tend to prioritize treatment of acute malnutrition. It was less common for wasting prevention to be mentioned as an explicit priority. Many interventions were perceived by respondents to influence both stunting and wasting despite limited published evidence of effectiveness on wasting for water, sanitation and hygiene, growth monitoring and promotion, breastfeeding promotion and micronutrient supplementation. To help unify the nutrition community around prevention of wasting, the discourse about priority interventions should shift from ‘stunting versus wasting’ and ‘prevention versus treatment’ to a life‐course framing around child survival, growth and development. Respondents identified a need for more programme and research funding that prioritizes both wasting and stunting as outcomes. They also suggest leveraging existing national coordination bodies that bring development and emergency‐humanitarian partners together.

**Key messages**

- Ongoing divisions of the nutrition community between development and emergency‐humanitarian actors hinder the prioritization of wasting prevention at global and country levels.
- Despite limited or weak evidence for their effectiveness on wasting, many nutrition professionals believe that water, sanitation and hygiene; growth monitoring and promotion; breastfeeding promotion; and micronutrient supplementation will influence wasting.
- For promoting wasting prevention, a life‐course framing that emphasizes child survival, growth and development is likely more effective than an outcome‐focused framing on stunting and/or wasting.
- Programme implementation and research initiatives as well as their funding institutions should include targets and metrics for both wasting and stunting.

## 1 INTRODUCTION

A range of interventions for the prevention and treatment of malnutrition in young children is being scaled up across countries with high burdens of undernutrition (Bhutta et al., 2013). Despite generalized support for combatting malnutrition, there has been ‘fragmentation of interests and perspectives’ within the global nutrition community related to which nutrition outcomes different camps of actors prioritize (Menon & Stoltzfus, 2012). Historically, childhood wasting—often called acute malnutrition—was the global picture of malnutrition. Images of famine in Ethiopia in the 1970s and 1980s (BBC, 2014) reinforced wasting as the predominant view of malnutrition, and it continues to be the main priority of the emergency‐humanitarian community. However, stunting—also referred to as chronic malnutrition—overtook wasting as the dominant priority of many development‐focused actors with the release and related advocacy around the 2008 Lancet Series on Maternal and Child Undernutrition (Bhutta et al., 2008; Leroy & Frongillo, 2019; Victora et al., 2008). These two groups, emergency‐humanitarian and development, differ in the frameworks used to characterize and respond to malnutrition problems (Wasting and Stunting Technical Interest Group, 2018). Stunting reduction efforts concentrate on prevention interventions among children under 2 years while wasting‐focused strategies prioritize screening and treatment of wasted children under 5 years using community‐based protocols (Kennedy, Branca, Webb, Bhutta, & Brown, 2015; Stewart, Iannotti, Dewey, Michaelsen, & Onyango, 2013).

The *prevention* of wasting in particular has received less attention than the prevention of stunting; however, the situation is evolving. The 2012 World Health Assembly nutrition targets include both stunting goals and wasting goals (World Health Organization, 2017). Health systems are scaling up screening and treatment of acute malnutrition as part of routine services (The Council of Research and Technical Advice on Acute Malnutrition, 2018). In addition, organizations like ‘No Wasted Lives’ are working to raise the profile of wasting in the global development agenda (The Council of Research and Technical Advice on Acute Malnutrition, 2018). Since 2014, the Emergency Nutrition Network (ENN) has coordinated the Wasting and Stunting (WaST) Technical Interest Group, which has advocated that more research and interventions need to be focused on identifying and addressing the common environmental and dietary risk factors and physiologic processes that contribute to both ponderal and linear growth faltering (Angood, 2014). These two conditions often co‐exist in the same populations of children (Khara, Mwangome, Ngari, & Dolan, 2018; Schoenbuchner et al., 2019; Uauy, Garmendia, & Corvalán, 2014; Wells et al., 2019).

Given common risk factors, it seems logical to assume that many interventions to prevent stunting will also prevent wasting (Briend, 2019; Myatt et al., 2018; Walson & Berkley, 2018). However, there is a complex and often oversimplified etiological relationship between wasting and stunting that still needs to be elucidated (Briend, 2019; Schoenbuchner et al., 2019). A recent review by ENN found that the current evidence base is not sufficient to conclude which interventions prevent wasting in non‐emergency contexts (Emergency Nutrition Network [ENN], 2019). In a review of evidence carried out for The Lives Saved Tool, 2020 (Panjwani & Heidkamp, 2017), members of our research team found that fewer studies of nutrition education interventions reported on wasting‐related outcomes as compared with food supplementation interventions. They also found that ponderal outcomes were less consistently reported across studies compared with linear growth. (Panjwani & Heidkamp, 2017). This raised questions about whether the variability in reported outcomes across studies also reflected differing priorities of development versus emergency‐humanitarian actors behind the studies (Ickes, Craig, & Heidkamp, 2019). To achieve global targets, new approaches are needed to bridge divides and to strengthen the overall evidence base for wasting prevention. Most policy and programme decision‐making is based on the perceptions of stakeholders (Pelletier, Menon, Ngo, Frongillo, & Frongillo, 2011; Tumilowicz et al., 2019). Therefore, it is important to understand how nutrition actors across various contexts currently perceive wasting, including its relative priority, what actions are effective, and whether there are competing interests within the communities who are addressing undernutrition. To this end, this study aimed to answer to two overarching questions: (a) How do global nutrition professionals and institutions perceive and prioritize prevention of wasting? (b) Do nutrition professionals identify different interest groups with competing priorities within their nutrition community? If so, how can divisions be overcome?

## 2 METHODS

We used several complementary approaches to answer the research questions including key informant interviews (KII), an online stakeholder survey and an analysis of nutrition‐focused strategic documents from organizations that work in low‐ and middle‐income countries (LMICs). Table S2 summarizes key terminology and definitions of nutritional status in children 0–59 months of age.

KII: In January–February 2018, KII were conducted with respondents from five focal countries (Bangladesh, Burkina Faso, India, Mozambique and Tanzania) and several research and development partner institutions with significant nutrition portfolios. The KII component was informed by a previous systematic review from the study team (Panjwani & Heidkamp, 2017) and a recent review of literature that examines the relationships between wasting and stunting (ENN, 2019). Focal countries were purposively selected from two high‐burden regions (sub‐Saharan Africa and Asia) based on a relatively high prevalence of stunting and wasting (Table SS1) and funder priorities. The sampling method was purposive in order to obtain information‐rich cases with expertise around nutrition policies and programmes in low‐resource contexts (Patton, 2002). The selected focal countries reflect the high rates of stunting and wasting in sub‐Saharan Africa and Asia, the two regions with the highest global burden (Alderman, Behrman, Glewwe, Fernald, & Walker, 2017). The authors adhered to the Consolidated Criteria for Reporting Qualitative Research (Table S3) (Tong, Sainsbury, & Craig, 2007). Eligible participants were identified by the researchers through professional networks, which included both academic researchers and key personnel at nongovernmental organizations (NGOs) working within or across the focal countries as well as snowball sampling within recruited respondents. Thirty eligible participants were recruited via email. Twenty‐one (70%) participated, and nine did not respond; however, none directly refused to participate. In one instance, two individuals from the same organization were interviewed.

The lead author (S. I.), a professor of nutrition with expertise in maternal and child nutrition in low‐income contexts, conducted all KII via phone in English. The interviewer had a prior collaborative relationship with two of the 21 KII participants. The average interview duration was 40 min. Interview participants were sent the interview guide and details on researcher's goals for the project via email in advance of the interview. Verbal informed consent was obtained prior to starting the interview. The interviews were audio recorded and transcribed verbatim. No repeat interviews were conducted. Interview transcripts were analysed in Dedoose qualitative analysis (Los Angeles, CA, 2018) by four trained research assistants using thematic codes. The research team developed the codebook collaboratively to ensure consistent code definitions were understood by all coders. The first two interviews were used as a test case to ensure that an interrater reliability of at least 90% was achieved. In the rare case of a coding ambiguity, researchers consulted with the lead author (S. I.). All interviews were double coded to ensure consistency of data classification. To illustrate key themes, rich text quotes were extracted along with de‐identified participant information.

A combined grounded theory and content analysis approach was used; codes were developed deductively from the interview guides and inductively from interview findings (Miles, Huberman, & Saldaña, 2014). Code reports were produced for each code to be analysed, and were then grouped according to five main topic families associated with the research questions. Fifteen initial codes (Table S4) were developed that were further grouped into four broader categories: (a) intervention types and categorization; (b) treatment of MAM and prevention of wasting; (c) factors used to target nutrition interventions; and (d) recognition of nutrition intervention ‘camps’ and ideas for building harmonization.

### 2.1 Stakeholder survey

Following the KII, an online survey was developed and disseminated using Qualtrics (Provo, UT, 2018) from April to May 2018. The aim of the survey was to capture the perspective of a wider range of respondents working across LMIC. Participants were invited to complete the survey using a mass email distribution or personal email invitation from the authors. Emails were sent to the following listservs: Accelerated Reduction Effort on Anemia, Ag2Nut, Core Group Nutrition and USAID SPRING Nutrition Budget and Planning. In addition, the 21 individuals who participated in KII were invited to participate in the online survey. Online survey recruitment began in late April and was closed in mid‐May 2018.

The online survey was developed from the findings of the KII and asked specifically which nutrition interventions are implemented in the respondent's context and whether the divisions described in the report introduction were apparent in their context. In addition, using a list of 20 interventions identified through KII, respondents identified which outcomes (wasting, stunting, both wasting and stunting or neither wasting nor stunting) they believed each intervention would influence. Respondents were asked an open‐response question about meaningful ways to categorize interventions. For the question about whether stunting and wasting are addressed by the same project or organizations in their context, participants were given three response options (agree a little, agree somewhat and agree a lot) but could not select ‘do not agree’, which was an oversight in survey design.

The survey was conducted in English. No sample size target was determined a priori, rather the researchers sought to obtain the largest sample possible within the specified time period. Participants provided written electronic consent; however, responses could not be linked to individual respondents due to lack of identifiers.

### 2.2 Policy and programme document review

Strategic documents from 12 institutions, including governments, UN agencies and NGOs working in child nutrition in LMIC were reviewed (**Box** **A**). At least one document was included from the government or nutrition‐focused UN institution in each of the five KII focal countries as well as Nigeria, a high burden country of interest to the funder. All documents were current as of February 2019. For each document, stated goals and prioritized indicators/outcomes were summarized. Multi‐national organizational documents were selected to represent leading organizations in global child nutrition programme funding and implementation. The aim of the review was to identify the types interventions planned or implemented among children 0 to 59 months and to assess how they describe and prioritize specific outcomes and interventions.

Box A **List of nutrition strategic plans included in the review**

**National organizations**

National Strategic Plan of Action for Nutrition (NSPAN) (2014‐2019)– Nigeria

National Multi‐sectoral Nutrition Action Plan (NMNAP) (2016‐2021) – Tanzania

National Nutrition Strategy (2018‐2022) – India

United Nations Agenda for the Reduction of Chronic Undernutrition in Mozambique (2015‐2019)

World Food Programme Nutrition Strategy (2017‐2020) ‐ Bangladesh

**Multi‐national organizations**

Bill & Melinda Gates Foundation Nutrition Strategy Overview (2019)

Catholic Relief Services Nutrition Program Description (2019)

Children's Investment Fund Foundation Strategy (2019)

Emergency Nutrition Network Strategy (2016‐2020)

International Rescue Committee Nutrition Program Description (2018)

United States Agency for International Development (USAID) Multisectoral Nutrition Strategy (2014‐2025)

World Food Programme Strategic Plan (2017‐2021)

Using a triangulation design (Creswell & Plano Clark, 2011), the research team analysed each data collection method independently and then integrated them under four common themes consistent with the research questions: (a) nutrition professionals' perception and prioritization of wasting as an outcome; (b) perception of outcomes associated with interventions; (c) evidence of divisions within the nutrition community; and (d) ideas for building harmonization across these divisions.

### 2.3 Ethical considerations

Wheaton College and Johns Hopkins University School of Public Health Institutional Review Boards approved the study procedures.

## 3 RESULTS

Twenty‐one KII were conducted between January and March 2018. Five participants were employed in UN agencies, nine were from NGOs, four were from governmental organizations and three were nutrition researchers employed at universities. Eleven of the participants were nationals of one of the focal countries. Five KII participants were primarily engaged in emergency‐humanitarian organizations or research, while the remainder worked for organizations that spanned both development activities and emergency‐humanitarian activities.

A total of 107 respondents participated in the online survey, of whom 75 completed all questions. The majority of respondents worked for NGOs (53%, *n* = 56), followed by government (16%, *n* = 17) and university or research institutions (18%, *n* = 20). Common professional responsibilities included programme planning, daily programme management, research and programme evaluation. About two‐thirds (64%, *n* = 68) of online survey respondents worked in a single country, and one‐third (36%, *n* = 39) worked across multiple countries. The three most common regions for work were sub‐Saharan Africa (44%, *n* = 47), South Asia (34%, *n* = 37) and Latin America/Caribbean/South America (16%, *n* = 17).

### 3.1 Perceptions and prioritization of wasting

Findings from all three data collection activities—KII, online survey and review of strategic documents—suggest that prevention of stunting is generally a higher priority objective than wasting prevention. Many KII national respondents said that wasting remains a critical nutrition problem. However, several suggested that global efforts to prioritize stunting prevention may contribute to de‐prioritization of wasting in their contexts. As one participant noted, ‘Some donors see stunting as a “bigger picture” than wasting’ (Tanzania). Another respondent noted: ‘[The prevention of stunting] has become a big, big objective in the world’ (Burkina Faso). Some participants noted that the lower prevalence of wasting may also reduce the incentive to address it: ‘Your reach in a wasting program is going to be a lot lower. Because not as many people are affected, it tends to be deprioritized a little bit’ (Multi‐country stakeholder, United States).

While participants understood the term ‘wasting prevention’ and felt it would be understood in their contexts, they said it was not commonly used.

One intervention I can think of on the health side is community case management. This kind of a preventive approach because you are trying to quickly get to a child at the community level with treatment for an illness which would then prevent the wasting that would come probably right after that incidence of illness. So, I think [wasting prevention] would be understood, but I really do not think we utilize it. And, whether it would be used outside of our community? You know there's been a lot more traction I would say in the general population around stunting and chronic malnutrition and how to prevent that but I think the wasting prevention may or may not stick with people (Multi‐country stakeholder, United Kingdom).

Our findings did not indicate any notable differences in views regarding wasting and stunting by geographic region or working within a single versus multi‐country setting.

The strategic document review highlighted differences in prioritization of approaches for wasting and stunting across government and development partner strategies. This analysis, summarized in Table 1, suggests that wasting prevention is a named priority, but it lags behind both wasting treatment and stunting prevention. Six institutions explicitly identified the prevention of wasting as a goal compared with eight that identified treatment of moderate acute malnutrition (MAM) and eight that identified prevention of stunting. Notably, among the 10 strategies that included monitoring and evaluations plans, stunting and wasting were both included as key performance indicators in seven plans.

**TABLE 1:** Organizational strategy analysis

Organization and name of strategy/programme	Stated goal			Classifications of interventions used	Prioritized outcomes/indicators
	Prevention of wasting	Treatment of MAM	Prevention of stunting		
Multi‐country					
Bill & Melinda Gates Foundation Nutrition Strategy Overview	No	No	No	Life course/1,000‐day; strengthening food systems	Prevention of nutrition‐related deaths; standardization of monitoring of nutrition data
Catholic Relief Services Nutrition Program Description	No	No	Yes	Life course	N/A
Children's Investment Fund Foundation Strategy	Yes	Yes	Yes	Delivery platform; business/marketplace	Prevalence of stunting (HAZ); prevalence of wasting (WHZ); prevalence of severe acute malnutrition (WHZ, MUAC)
Emergency Nutrition Network Strategy (2016–2020)	Yes	No	No	Evidence‐based interventions	Prevalence of wasting, SAM, and MAM, stunting and wasting (relationships); factors associated with wasting
International Rescue Committee Nutrition Program Description	No	Yes	No	Acute malnutrition; IYCF practices	N/A
United States Agency for International Development (USAID) Multisectoral Nutrition Strategy (2014–2025)	No	Yes	Yes	Chronic versus acute; nutrition specific & nutrition sensitive.	Prevalence of stunting (HAZ); prevalence of wasting (WHZ); prevalence of SAM (WHZ, MUAC)
World Food Programme Strategic Plan (2017–2021)	No^a^	No	Yes	Chronic versus acute; nutrition specific & nutrition sensitive.	Prevalence of stunting (HAZ); prevalence of wasting (WHZ)
Country specific					
National Strategic Plan of Action for Nutrition (2014–2019)–Nigeria	Yes	Yes	Yes	Various (sector, life course, etc.)	Prevalence of: Under 5 (U5) stunting (HAZ); LBW, U5 wasting (WHZ); women 15–49 with anaemia; children EBF < 6 m.
National Multi‐sectoral Nutrition Action Plan (2016–2021)–Tanzania	Yes	Yes	Yes	Nutrition specific & nutrition sensitive; enabling environment.	Prevalence of: U5 stunting (HAZ); anaemia in women; LBW; rate of EBF (0 < 6 months); U5 wasting; U5 vitamin A deficiency; U5 underweight; U5 anaemia; iodine deficiency
National Nutrition Strategy (2018–2022)–India	Yes	Yes	No	Life course × platform (community nutrition)	Prevalence of U5 underweight; prevalence of U5 anaemia; prevalence of anaemia in mothers of reproductive age
United Nations Agenda for the Reduction of Chronic Undernutrition in Mozambique (2015–2019)	No	No	Yes	Various (sector, life course, etc.)	Prevalence of stunting (HAZ); prevalence of acute malnutrition (WHZ)
World Food Programme Nutrition Strategy (2017–2020)–Bangladesh	Yes	Yes	Yes	Nutrition specific & nutrition sensitive	Prevalence of malnutrition (general), prevalence of low birth weight

Abbreviations: MAM, moderate acute malnutrition; MUAC, middle‐upper arm circumference; N/A, Not Applicable; SAM, severe acute malnutrition; WHZ, weight‐for‐height *Z* score; WLZ, weight‐for‐length *Z* score; HAZ, Hieght‐for‐age z score; EBF, exclusive breastfeeding; LBW, low birth weight; SDG, Sustainable Development Goals.

^a^ Prevention of wasting not explicitly stated but implied in organizational alignment with SDG.

### 3.2 Views on effective interventions for wasting

**FIGURE 1:**
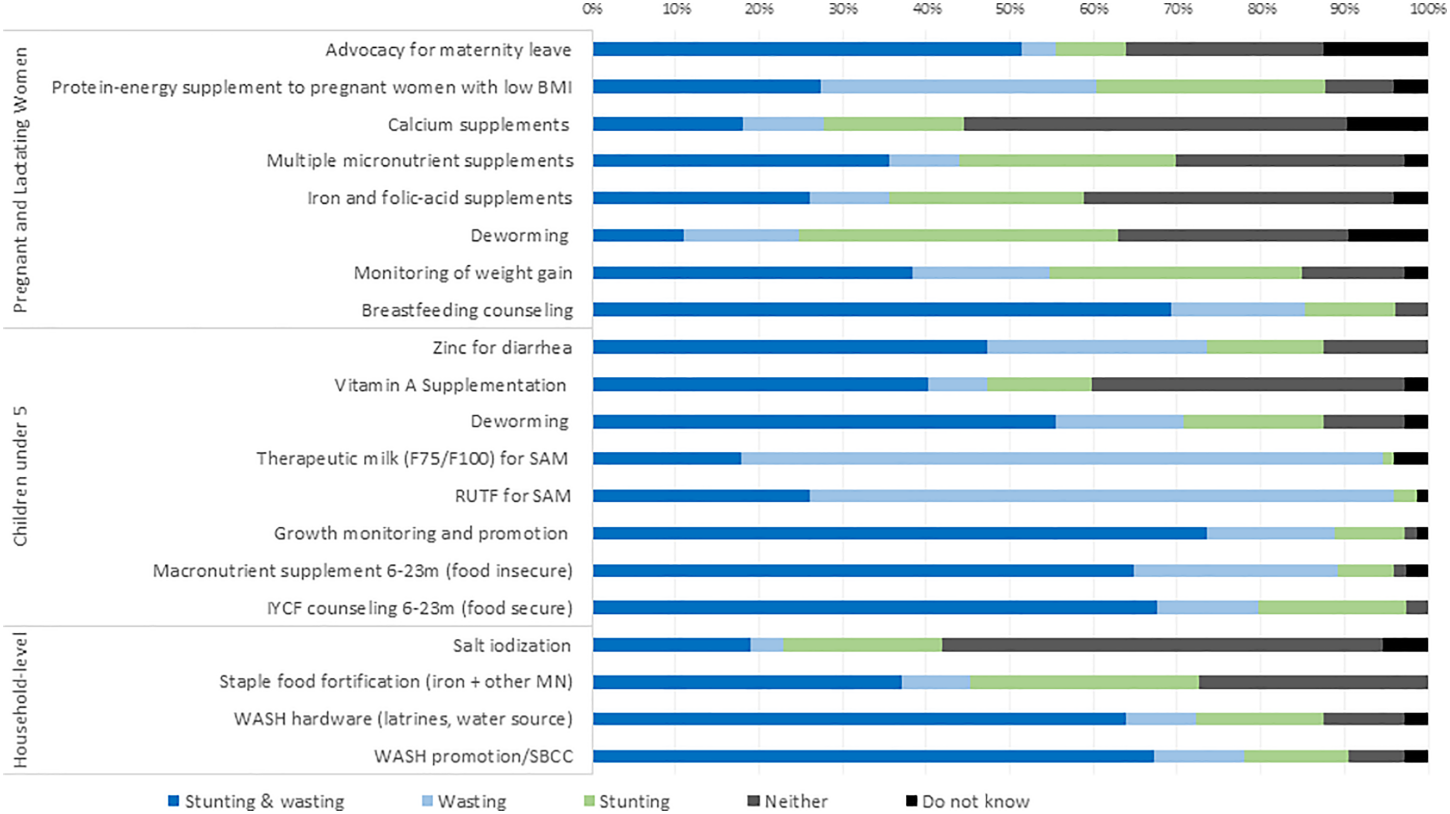
Online survey respondents' categorization of maternal and child nutrition interventions by outcome (*n* = 107). BMI, body mass index; SAM, severe acute malnutrition; WASH, water, sanitation and hygiene

Figure 1 summarizes responses from online survey respondents asked to categorize a list of interventions according to which outcomes they influence. Only severe acute malnutrition (SAM) treatment interventions were predominantly associated with wasting only. Most respondents identified several interventions including growth monitoring and promotion (74%, *n* = 79), breastfeeding counselling (69%, *n* = 74), Infant and Young Child Feeding (IYCF) counselling in food‐secure settings (68%, *n* = 73), water, sanitation and hygiene (WASH) promotion and social behaviour change communication (67%, *n* = 72), and macronutrient supplementation in food‐insecure settings (66%, *n* = 71) as having impact on both wasting and stunting.

KII also showed that stakeholders associate certain interventions like IYCF promotion with influencing multiple outcomes:

We are stunting focused, but address wasting and the most common micronutrient deficiency: anaemia. When you make a very good promotion of IYCF practices, I think you have impact on stunting reduction, anaemia reduction, and also wasting reduction (Single‐country stakeholder, Burkina Faso).

In Table 2, the six interventions that online survey respondents most commonly associated with wasting (either alone or with stunting) are compared with the evidence for their effectiveness as summarized in a recent ENN review (ENN, 2018).

**TABLE 2:** Current evidence base and effectiveness of wasting prevention interventions for online survey respondent's recommended interventions for wasting prevention

Interventions identified in KII as ‘suitable for wasting prevention’	Strength of the body of evidence for this intervention^a^	Evidence of effectiveness for preventing wasting^b^
Nutrition education or counselling	Medium/limited number of quality studies	Modest effects observed in some trials/effects found, but in poor‐quality trials
WASH improvements	Medium/limited number of quality studies	No significant effects found
Macronutrient/food supplementation (includes MAM treatment)	Several quality studies (including systematic reviews, RCTs)	Significant effects observed on wasting prevention in several studies
Micronutrient supplementation	Several quality studies (including systematic reviews, RCTs)	No significant effects found
Breastfeeding promotion	Medium/limited number of quality studies	No significant effects found
Multi‐sectoral or combination interventions	Very few or no quality studies	Modest effects observed in some trials/effects found but in poor‐quality trials

Abbreviation: KII, key informant interviews; MAM, moderate acute malnutrition; RCT, randomized controlled trial; WASH, water, sanitation and hygiene.

^a^ Green = several quality studies (including systematic reviews, RCTs); Yellow = medium/limited number of quality studies; Red = very few or no quality studies.

^b^ Green = significant effects observed on wasting prevention in several studies; Yellow = modest effects observed in some trials/effects found but in poor‐quality trials; Red = no significant effects found. Source: Emergency Nutrition Network. The Current State of Evidence and Thinking on Wasting Prevention. 2018.

For food supplementation interventions, stakeholder responses align well with evidence. However, a high proportion of respondents identified WASH, growth monitoring and promotion, breastfeeding promotion and micronutrient supplementation as suitable to affect wasting. The evidence base contradicts this, is weak, or does not show strong significant effects. It should be noted that the ENN review (ENN, 2018) did not include growth monitoring and promotion.

For the micronutrient interventions there were notable differences between respondents who worked across multiple countries (*n* = 27) compared with those working in a single country (*n* = 48). Single‐country respondents were more likely to identify multiple micronutrient supplements (MMS) during pregnancy as having impact on both stunting and wasting (45%, *n* = 22 of single‐country versus 19%, *n* = 5 of multi‐country). More than half of multi‐country respondents (54%, *n* = 15) selected ‘neither outcome’ for MMS compared with 13% of single‐country respondents (*n* = 6). There was a similar pattern of selecting ‘neither outcome’ for iron and folic acid supplementation during pregnancy (62%, *n* = 17 of multi‐country vs. 23%, *n* = 11 of single country) and for high‐dose vitamin A supplementation in children (64%, *n* = 17 of multi‐country vs. 23%, *n* = 11 of single country). The subgroup differences may be attributable to multi‐country stakeholders from UN agencies, donors or multinational NGOs having more access to global evidence reviews than those focused on single countries who are more likely to work for government or local NGOs.

#### 3.2.1 Perceptions of SAM versus MAM treatment

Some KII saw the potential for ‘treatment of MAM’ to prevent SAM—but several considered this an impractical and unsustainable strategy given the low coverage and high cost relative to the burden.

One participant said, ‘Lots of programs combine bits of both so that MAM programs are [a type of] primary prevention for SAM … There's a big overlap [in MAM and SAM] and these treatments are not an either‐or but very much a both‐and. In preventing one thing, or treating one thing [you] can prevent another’ (Multi‐country, United Kingdom).

A KII respondent in Tanzania said the country has clear protocols for SAM but not for MAM treatment. A key informant in Bangladesh and India indicated that the incorporation of MAM treatment into routine health services was not possible due to capacity constraints and was ‘unsustainable’, especially for already overburdened local health centres and the high cost of MAM treatment. This respondent described a ‘massive gap in coverage’ for MAM treatment due the cost of a national community‐based management of acute malnutrition and a resistance to specialized products for addressing MAM:

There is huge resistance to [lipid‐based nutrient supplements] and [ready‐to‐use therapeutic food]. There are multiple dimensions to that resistance. I think that [national stakeholders] see it as sort of a multi‐national product‐driven approach. It can be taking time and resources away from programming that could be developed nationally and much more focused on improving quality of home‐based food and we cannot argue against that because there is a huge amount of poverty. It's not like people are not trying to do better, but that's their argument (Multi‐country stakeholder, India and Bangladesh).

Evidence of division within the nutrition community.

Online survey and KII respondents largely agreed that ‘stunting prevention’ and ‘wasting treatment and prevention’ involve different actors for funding and implementation. They had different explanations for why the divide occurred.

Yes, I think [the development of camps] is not deliberate. I guess I see it just as a part of the history or particularly [community‐based management of acute malnutrition] in the humanitarian world where the rapid response programs were priority and I think that that's why it has been scaled up so quickly. But the flip side is that it's perhaps not integrated. I see lots more programs working to make that happen now, and lots more recognition that that's needed. So, the conversations are happening and lots of places are doing things to impact. I think it needs to continue. We need to keep talking and working through the details (Multi‐country stakeholder, UK).

The reason that stunting and wasting diverged as forms of malnutrition if you want to go back 50 years is because one responded to food and the other one did not. So, people took wasting out and said this is one we can do something about. Let us label this and promote programs along these lines and then stunting that does not respond to food and does not seem to respond to bed nets that reduce malaria transmission by 10‐fold, and you know very excellent dietary supplements. Even in the last year, stunting does not really respond to sanitation interventions. Well, what is stunting, it is not responsive to these particular things. The flaw in that kind of thinking is that, the challenge is that stunting is a multifaceted process and the interventions that are going to reduce it will have to be multifaceted. So, that is kind of my view of the landscape (Multi‐country stakeholder, United States).

The online survey findings indicated a lack of strong agreement about whether the same projects and organizations address both stunting and wasting in stakeholder contexts. Only 35% agreed strongly with the statement that ‘the same projects address both stunting and wasting’ (*n* = 37, 35%). Fewer (*n* = 22, 21%) agreed strongly that ‘the same organizations are responsible for planning and implementing projects to address both wasting and stunting.’

### 3.3 Strategies for bridging divisions within the nutrition community

Stakeholders suggested different approaches for bridging the divide between development and emergency‐humanitarian groups. Those working across multiple countries advocated for greater harmonization in funding sources and reframing discourse and terminology around wasting. Respondents working in single countries emphasized improvements in national coordination.

#### 3.3.1 Reframing discourse and terminology

Within the organizational strategic plans, the most common classifications used to describe intervention activities were using the life course (pregnancy, infancy, early childhood and adolescence) and ‘nutrition sensitive versus nutrition specific’ (Table 1).

Among survey participants, 41 suggested an alternative categorization to ‘stunting versus wasting focused’ in response to an open‐ended question. The most common suggestions were life course or age‐based (*n* = 11, 27%), ‘prevention versus treatment’ (*n* = 9, 22%) and by the nutritional status being targeted (e.g., micronutrient deficiency and chronic undernutrition) (*n* = 9, 22%). Participants also suggested grouping interventions according to whether they achieve intermediate or long‐term outcomes (*n* = 2, 5%), the delivery platform of the intervention (*n* = 1, 2%), or more generally ‘targeting’ (*n* = 8, 20%).

Integration of services and harmonization of priority outcomes towards the common goal of a well‐nourished or healthy child was a repeated theme. Reframing the discourse was seen as a strategy to this end:

I think we need to change how we talk about child growth. We need to stop talking about stunting versus wasting and just talk about child growth in general. So I think that is something to think about in the community because I think it might help move this discussion forward, and I think we need to have a better understanding of risks, too, because I know that a child that is SAM or MAM has a really high risk of mortality. For those that are chronically malnourished and stunted, I think we do not communicate enough about their risks. Like a child that is very, very low HAZ, they also have a high mortality risk at well, and I think it's even more than MAM kids. But, we need to remember that these kids are generally at risk here and we need to be doing something about it. So I think our language and how we talk about child growth could be one thing (Multi‐country stakeholder, Canada). Moreover, stakeholders noted a recent shift in the conversation and increase in programming that is inclusive of wasting and stunting.

We have created all these sort of artificial divisions for programmatic reasons, you know like SAM and MAM, wasting, stunting. But, they are all occurring in the same children, and the wasting‐stunting overlap is massive and you know we furthermore know that children are both wasted and stunted have the highest risk of mortality. So, there is a lot of talk recently, even kind of blurring those lines even more. In terms of special definitions that we are talking about when we talk about undernourished children also linking with traditional prevention programs like cash transfers, WASH … absolutely. At [our organization] we are working on integrating packages, every agency does (Multi‐country stakeholder, USA).

#### 3.3.2 Improved strategies for coordination and funding

Stakeholders identified government as a key institution for coordination of different stakeholder groups. Specific examples including the Scaling Up Nutrition (SUN) movement in Tanzania were seen as helping to bring together nutrition professionals working across the emergency‐humanitarian and development spectrum. Efforts to bring together government with academic and the private sector were also noted as important.

KII respondents provided a number of examples of national‐level coordination strategies that can help bridge implementation divides.

You know at the global level you have some of these clusters—the nutrition cluster, the health cluster, and things like that— where UNICEF tends to manage the nutrition cluster. But I think within countries is really where you need to start, and you need to have a sort of goal there, and you need to have a national nutrition policy that brings it all together as opposed to one that might be a little bit fracture (Multi‐county stakeholder, USA).

All these groups get together on a quarterly basis. [We need to] look at the specifics: what are they called? What they do plan? How do we move forward? … All these have been brought to them at the secretariat group within the nutrition sector. So, actually I think it's an example to … other sectors because we are well coordinated [in the nutrition sector]. We've picked the same language, we are serving the same plan, so we are following you wherever you are working in this big country. You have a road map to follow (Single‐country stakeholder, Tanzania).

One multi‐country stakeholder identified Kenya and Ethiopia as countries where national‐level coordination is taking place and the same actors are involved in wasting and stunting activities.

Kenya comes to mind … I feel like the people on the ground that are actually implementing nutrition programming are actually the same people that are scaling up their treatment for wasting in emergencies, and they are the same people that are investing in chronic malnutrition and stunting … Ethiopia would be another with the coordination mechanism. But, there is … still room for improvement on that (Multi‐country stakeholder, Canada).

Professional communities of practice and their associated listservs were also noted as a way to ‘break down some of the barriers and convene diverse groups beyond what was possible with physical meetings’ (Multi‐country stakeholder, United States).

Funding and technical expertise needed to address nutrition problems were cited as barriers to greater harmonization.

I think the bottom line though is funding, because it would be wonderful to work at the multi‐sectoral level, but it takes huge amounts of funding to implement those integrated packages … Donor money is not there for that (Multi‐country stakeholder, Kenya).

Stakeholders noted that the breadth and complexity of nutrition make ‘developing one organization that understands the entire context and is able to program very well’ difficult, suggesting that organization expertise is needed alongside inter‐organizational coordination (Multi‐country stakeholder, USA).

## 4 DISCUSSION

This study's findings confirm that despite a global target to reduce wasting (WHO/UNICEF/WFP, 2014), wasting prevention is not being prioritized relative to stunting prevention or wasting treatment. ‘Humanitarian’ versus ‘development’ divisions are recognized at global and country levels and generally seen to be counterproductive to achieving common goals including the scale‐up of interventions to address both wasting and stunting. However, respondents identified several approaches for overcoming divisions including adopting terminology that focused on more holistic objectives and advocacy for funding that links wasting‐ and stunting‐related programming. Leveraging existing national coordination bodies such as those promoted by the SUN movement (e.g., national multi‐sector committees and civil society networks) or the Intra‐Agency Standing Committee Cluster Approach was another strategy with potential to bring development and emergency‐humanitarian together partners together.

The current gap between stakeholder perception and rigorous evaluation evidence about what strategies are effective for wasting and stunting may cause investments to be misdirected and nutrition outcomes to be suboptimal. Without clear evidence for intervention impact on wasting prevention, it is difficult to make a case to prioritize them. Harmonization of study design—particularly around duration and indicators assessed—are needed to capture declines in wasting incidence and prevalence (ENN, 2019; Ickes et al., 2019). While not included in our survey, we also need evidence for the effect of nutrition‐sensitive interventions that seek to influence the underlying determinants of undernutrition through agricultural health, social protection, early childhood development, education and water and sanitation (Cordon, Asturias, Vries, & Rohloff, 2019; Ruel & Alderman, 2013).

The need for stunting and wasting to both be prioritized by governments, donors and implementing partners is echoed by a recent commentary by Leroy and Frongillo (2019) that critiques the prioritization of stunting. The authors advocate for using outcomes that are more proximal to interventions and inclusive of other dimensions of child wellbeing.

### 4.1 Strengths and limitations

The primary strength of this study is the application of multiple methods to obtain the perspectives of a wide range of nutrition stakeholders including those working within single countries and across multiple countries, who often have different priorities. This study also had several limitations. First, the online survey did not require responses to all questions and therefore had limited responses to select questions. Second, the study may have been affected by selection bias due to non‐response, whereby more motivated or invested respondents chose to participate. Individuals with lower access to the internet may have been less likely to participate in the online survey or a Skype interview. In an effort to mitigate telecommunications issues, interviewees were called by phone directly when platforms such as Skype were problematic for participants, and interviews were scheduled during the most convenient times for the participants. Both KII and online surveys were only conducted in English, which may have biased the responses received. Third, information bias may have been introduced due to the omission of a ‘do not agree’ option for the two survey questions that asked if the same projects and organizations address both stunting and wasting in participant contexts. While bias is possible from this design oversight, if respondents truly did not agree, the logical alternate selection would have been to ‘agree a little’ versus to ‘agree somewhat’ or ‘a lot’. Given that the majority of participants did not ‘agree a lot’ with these two questions, any information bias that resulted from this design flaw appears to be minimal.

## 5 CONCLUSION

The burden of multiple forms of malnutrition remains high in many countries. One in five children is stunted (Global Nutrition Report, 2018). An estimated 3% of children globally—about 6 million children—are both stunted and wasted and face a high risk of mortality (Khara et al., 2018). Of the 184 countries assessed in the most recent Global Nutrition Report, only 18 are on track to meet both World Health Assembly wasting and stunting targets by 2025. Divisions within the nutrition community and misconceptions around the impact of interventions are only two of several factors contributing to slow progress in addressing undernutrition. However, they are factors that can and must be addressed through advocacy and coordinated investments in research.

## CONFLICTS OF INTEREST

The authors declare that they have no conflicts of interest.

## CONTRIBUTIONS

SI, CC and RH performed the research. RH and SI designed the research study. SI, RH and CC analysed the data and wrote the paper. All authors read and approved the final manuscript.

## Supporting information


**Table S1**. Stunting and wasting prevalence estimates in the five project focal countries
**Table S2**. Terminology and definitions of nutritional status in children 0–59 months of age
**Table S3**. COREQ Criteria (uploaded as a PDF).
**Table S4**. Codebook for key informant interview analysis

## References

[mcn13035-bib-0001] Alderman, H. , Behrman, J. R. , Glewwe, P. , Fernald, L. , & Walker, S. (2017). Evidence of impact of interventions on growth and development during early and middle childhood In Bundy D. A. P., de Silva N., Horton S., Jamison D. T., & Patton G. C. (Eds.), Child and adolescent health and development (3rd ed.). Washington (DC): The International Bank for Reconstruction and Development/The World Bank Retrieved from http://www.ncbi.nlm.nih.gov/books/NBK525234/, DOI: 10.1596/978-1-4648-0423-6_ch7 30212122

[mcn13035-bib-0002] Angood, C . (2014). The wasting and stunting (WaSt) project – Investigating the relationship between wasting and stunting and exploring the implications for policy programmes and research. Retrieved March 23, 2020, from www.ennonline.net/ourwork/reviews/wastingstunting

[mcn13035-bib-0003] BBC . (2014, October 24). Ethiopia's famine: Remembering 30 years on – BBC News. [Video file]. Retrieved from https://www.youtube.com/watch?v=6SVByPiF6iQ&feature=youtu.be

[mcn13035-bib-0004] Bhutta, Z. A. , Ahmed, T. , Black, R. E. , Cousens, S. , Dewey, K. , Giugliani, E. , … for the Maternal and Child Undernutrition Study Group . (2008). What works? Interventions for maternal and child undernutrition and survival. Lancet, 371, 417–440. 10.1016/S0140-6736(07)61693-6 18206226

[mcn13035-bib-0005] Bhutta, Z. A. , Das, J. K. , Rizvi, A. , Gaffey, M. F. , Walker, N. , Horton, S. , … Black, R. E. (2013). Evidence‐based interventions for improvement of maternal and child nutrition: What can be done and at what cost? The Lancet, 382(9890), 452–477. 10.1016/S0140-6736(13)60996-4 23746776

[mcn13035-bib-0006] Bill & Melinda Gates Foundation Nutrition Strategy Overview . (2019). Retrieved from https://www.gatesfoundation.org/What-We-Do/Global-Development/Nutrition

[mcn13035-bib-0008] Briend, A. (2019). The complex relationship between wasting and stunting. American Journal of Clinical Nutrition., 110(2), 271–272. 10.1093/ajcn/nqz050 31172176

[mcn13035-bib-0009] Catholic Relief Services Nutrition Program Description . (2019). Retrieved from https://www.crs.org/our-work-overseas/program-areas/nutrition

[mcn13035-bib-0010] Children's Investment Fund Foundation Strategy . (2019). Retrieved from https://ciff.org/priorities/childhood-adolescence/nutrition/

[mcn13035-bib-0011] Cordon, A. , Asturias, G. , Vries, T. D. , & Rohloff, P. (2019). Advancing child nutrition science in the scaling up nutrition era: A systematic scoping review of stunting research in Guatemala. BMJ Paediatrics Open, 3(1), e000571 10.1136/bmjpo-2019-000571 32099904 PMC7015046

[mcn13035-bib-0012] Creswell, J. W. , & Plano Clark, V. L. (2011). Designing and conducting mixed methods research (2nd ed.). London: Sage Publications Ltd.

[mcn13035-bib-1000] Emergency Nutrition Network . (2018). The Current State of Evidence and Thinking on Wasting Prevention. Available at: https://www.ennonline.net/fex/59/wastingprevention

[mcn13035-bib-0013] Emergency Nutrition Network . (2019). The current state of evidence and thinking on wasting prevention. Field Exchange 59, p29. Retrieved from. http://www.ennonline.net/fex/59/wastingprevention

[mcn13035-bib-0014] Emergency Nutrition Network Strategy . (2016‐2020). Retrieved from https://www.ennonline.net/attachments/2461/Strategy-2016-2020_WEB-final.pdf

[mcn13035-bib-0015] Global Nutrition Report . (2018). Retrieved from https://globalnutritionreport.org/reports/global-nutrition-report-2018. Submitted to the Children's Investment Fund Foundation.

[mcn13035-bib-0016] Ickes, S. , Craig, C. , & Heidkamp, R. (2019). Bringing prevention of wasting into nutrition community priorities: Nutrition stakeholder perspectives and systematic review of nutrition education and food supplementation interventions.

[mcn13035-bib-0017] International Rescue Committee Nutrition Program Description . (2018). https://www.rescue.org/resource/nutrition‐international‐rescue‐committee

[mcn13035-bib-0018] Kennedy, E. , Branca, F. , Webb, P. , Bhutta, Z. , & Brown, R. (2015). Setting the scene: An overview of issues related to policies and programs for moderate and severe acute malnutrition. Food and Nutrition Bulletin, 36(1 Suppl), S9–S14. 10.1177/15648265150361S102 25902609

[mcn13035-bib-0019] Khara, T. , Mwangome, M. , Ngari, M. , & Dolan, C. (2018). Children concurrently wasted and stunted: A meta‐analysis of prevalence data of children 6–59 months from 84 countries. Maternal & Child Nutrition, 14(2), e12516 10.1111/mcn.12516 28944990 PMC5901398

[mcn13035-bib-0020] Leroy, J. , & Frongillo, E. (2019). Perspective: What does stunting really mean? A critical review of the evidence. Advances in Nutrition, 10(2), 196–204. 10.1093/advances/nmy101 30801614 PMC6416038

[mcn13035-bib-0021] Menon, P. , & Stoltzfus, R. J. (2012). Building convergence in science, programs, and policy actions on child undernutrition: Symposium rationale and overview. Advances in Nutrition, 3(2), 224–226. 10.3945/an.111.001115 22516732 PMC3648725

[mcn13035-bib-0022] Miles, M. B. , Huberman, A. M. , & Saldaña, J. (2014). Qualitative data analysis: A methods sourcebook (3rd ed.). London, UK: SAGE.

[mcn13035-bib-0023] Myatt, M. , Khara, T. , Schoenbuchner, S. , Pietzsch, S. , Dolan, C. , Lelijveld, N. , & Briend, A. (2018). Children who are both wasted and stunted are also underweight and have a high risk of death: A descriptive epidemiology of multiple anthropometric deficits using data from 51 countries. Arch Public Health, 76, 28 10.1186/s13690-018-0277-1 30026945 PMC6047117

[mcn13035-bib-0024] National Multi‐sectoral Nutrition Action Plan (NMNAP) – Tanzania . (2016–2021). Retrieved from http://tfnc.go.tz/uploads/pressreleases/sw1520878189-NMNAP%202016-21.pdf

[mcn13035-bib-0025] National Nutrition Strategy – India . (2018‐2022) http://niti.gov.in/writereaddata/files/document_publication/Nutrition_Strategy_Booklet.pdf

[mcn13035-bib-0026] National Strategic Plan of Action for Nutrition (NSPAN) – Nigeria . (2014‐2019). Retrieved from https://extranet.who.int/nutrition/gina/en/node/23587

[mcn13035-bib-0027] Panjwani, A. , & Heidkamp, R. (2017). Complementary feeding interventions have a small but significant impact on linear and ponderal growth of children in low‐ and middle‐income countries: A systematic review and meta‐analysis. The Journal of Nutrition, 147(11), 2169S–2178S. 10.3945/jn.116.243857 28904113

[mcn13035-bib-0028] Patton, M. Q. (2002). Qualitative research and evaluation methods (3rd ed.). Thousand Oaks, CA: Sage Publications.

[mcn13035-bib-0029] Pelletier, D. L. , Menon, P. , Ngo, T. , Frongillo, E. A. , & Frongillo, D. (2011). The nutrition policy process: The role of strategic capacity in advancing national nutrition agendas. Food and Nutrition Bulletin, 32(2 Suppl), S59–S69. 10.1177/15648265110322S203 21916115

[mcn13035-bib-0030] Ruel, M. T. , & Alderman, H. (2013). Nutrition‐sensitive interventions and programmes: How can they help to accelerate progress in improving maternal and child nutrition? The Lancet, 382(9891), 536–551. 10.1016/S0140-6736(13)60843-0 23746780

[mcn13035-bib-0031] Schoenbuchner, S. M. , Dolan, C. , Mwangome, M. , Hall, A. , Richard, S. A. , Wells, J. C. , … Moore, S. E. (2019). The relationship between wasting and stunting: A retrospective cohort analysis of longitudinal data in Gambian children from 1976 to 2016. The American Journal of Clinical Nutrition, 110(2), 498–507. 10.1093/ajcn/nqy326 30753251 PMC6669055

[mcn13035-bib-0032] Stewart, C. P. , Iannotti, L. , Dewey, K. G. , Michaelsen, K. F. , & Onyango, A. W. (2013). Contextualizing complementary feeding in a broader framework for stunting prevention. Maternal and Child Nutrition, 9(Suppl 2), 26–45. 10.1111/mcn.12088 PMC6860787 24074316

[mcn13035-bib-0033] The Council of Research and Technical Advice on Acute Malnutrition . (2018). A research agenda for acute malnutrition (CORTASAM). No Wasted Lives; 2018.

[mcn13035-bib-0034] The Lives Saved Tool . (2020). The lives saved tool. Retrieved March 1, 2020, from https://www.livessavedtool.org

[mcn13035-bib-0035] Tong, A. , Sainsbury, P. , & Craig, J. (2007). Consolidated criteria for reporting qualitative research (COREQ): A 32‐item checklist for interviews and focus groups. International Journal for Quality in Health Care, 19, 349–357. 10.1093/intqhc/mzm042 17872937

[mcn13035-bib-0036] Tumilowicz, A. , Ruel, M. T. , Pelto, G. , Pelletier, D. , Monterrosa, E. C. , Lapping, K. , … Society for Implementation Science in Nutrition . (2019). Implementation science in nutrition: Concepts and frameworks for an emerging field of science and practice. Current Developments in Nutrition, 3(3), nzy080 10.1093/cdn/nzy080 30864563 PMC6400593

[mcn13035-bib-0037] Uauy, R. , Garmendia, M. L. , & Corvalán, C. (2014). Addressing the double burden of malnutrition with a common agenda. Nestle Nutrition Institute Workshop Series, 78, 39–52. 10.1159/000354935 24504205

[mcn13035-bib-0038] United Nations Agenda for the Reduction of Chronic Undernutrition in Mozambique . (2015‐2019). http://scalingupnutrition.org/wp‐content/uploads/2016/02/UN‐Agenda‐for‐the‐Reduction‐of‐Chronic‐Undernutrition‐Mozambique.pdf

[mcn13035-bib-0039] United States Agency for International Development (USAID) Mulitsectoral Nutrition Strategy . (2014‐2025). https://www.usaid.gov/nutrition‐strategy

[mcn13035-bib-0040] Victora, C. G. , Adair, L. , Fall, C. , Hallal, P. C. , Martorell, R. , Richter, L. , … Maternal and Child Undernutrition Study Group . (2008). Maternal and child undernutrition: Consequences for adult health and human capital. Lancet, 371, 340–357. 10.1016/S0140-6736(07)61692-4 18206223 PMC2258311

[mcn13035-bib-0041] Walson, J. L. , & Berkley, J. A. (2018). The impact of malnutrition on childhood infections. Current Opinion in Infectious Diseases, 31(3), 231–236. 10.1097/QCO.0000000000000448 29570495 PMC6037284

[mcn13035-bib-0042] Wasting‐Stunting Technical Interest Group . (2018). Child wasting and stunting: Time to overcome the separation. Emergency Nutrition Network.

[mcn13035-bib-0043] Wells, J. C. , Briend, A. , Boyd, E. M. , Berkely, J. A. , Hall, A. , Isanaka, S. , … Dolan, C. (2019). Beyond wasted and stunted—A major shift to fight child undernutrition. The Lancet Child & Adolescent Health, 3(11), 831–834. 10.1016/S2352-4642(19)30244-5 31521500

[mcn13035-bib-0044] WHO/UNICEF/WFP . (2014). Global nutrition targets 2025: Wasting policy brief (WHO/NMH/NHD/14.8). Geneva: World Health Organization.

[mcn13035-bib-0045] World Food Programme Nutrition Strategy – Bangladesh . (2017‐2020). Retrieved from https://docs.wfp.org/api/documents/WFP-0000019637/download/?_ga=2.208061584.320611093.1550786541-44927734.1550786541

[mcn13035-bib-0046] World Food Programme Strategic Plan . (2017‐2021). Retrieved from https://docs.wfp.org/api/documents/WFP-0000019573/download/?_ga=2.131273039.1373492103.1528794361-1767849330.1498626707

[mcn13035-bib-0047] World Health Organization . (2017). Global nutrition monitoring framework: Operational guidance for tracking progress in meeting targets for 2025.

