# Supplementary material for: How do nutrition professionals working in low‐income countries perceive and prioritize actions to prevent wasting? A mixed‐methods study
Source: Matern Child Nutr. 2020 Jun 8;16(4):e13035. doi: 10.1111/mcn.13035 (PMC7507008; doi:10.1111/mcn.13035)
Supplement: Supplementary file 1 — Table S1. Stunting and wasting prevalence estimates in the five project focal countries Table S2. Terminology and definitions of nutritional status in children 0–59 months of age Table S3. COREQ Criteria (uploaded as a PDF). Table S4. Codebook for key informant interview analysis [file MCN-16-e13035-s001.docx]

**Supplemental Table 1**. Stunting and wasting prevalence estimates in the five project focal countries

| **Country** | **Year/Source** | **U5 Stunting Prevalence (%)**  **(HAZ<-2 SD)** | **U5 Wasting Prevalence (%)**  **(WHZ<-2 SD)** |
| --- | --- | --- | --- |
| Bangladesh^^[[1]](#footnote-1)^^ | 2014 DHS | 36.1 | 14.3 |
| Burkina Faso ^^[[2]](#footnote-2)^^ | 2016 NNS (SMART) | 27.3 | 7.6 |
| India ^^[[3]](#footnote-3)^^ | 2015-2016 NFHS | 38.4 | 21.0 |
| Mozambique ^1^ | 2011 DHS | 42.6 | 5.9 |
| Tanzania^1^ | 2015-2016 DHS | 34.4 | 4.4 |

*Abbreviations*: DHS: Demographic and Health Survey; NNS; National Nutrition Survey; NNHS: National Nutrition and Health Survey; Data from Demographic and Health Survey STAT Compiler, NFHS, National Family Health Survey; National Nutrition Surveys (SMART), U5: under five years

| **Supplemental Table 2. Terminology and definitions of nutritional status in children 0-59 months of age**  **Wasted**           Moderate or severe: Weight-for-height < -2 SD of the WHO Child Growth Standard median           Severe: -3 SD of the WHO Child Growth Reference median  **Acute malnutrition**          Moderate or severe: Weight-for-height < -2 SD of the WHO Child Growth Standard median          MUAC < 125 mm          Bilateral pedal edema  **Severe Acute malnutrition**:  Weight-for-height < -3 SD of the WHO Child Growth Standard median          MUAC < 115 mm          Bilateral pedal edema    **Stunted**          Weight-for-height < -3 SD of the WHO Child Growth Standard median          MUAC < 115 mm          Nutritional edema (kwashiorkor) |
| --- |

Abbreviations: MUAC, middle-upper arm circumference; SD, standard deviation; WHO, World Health Organization. Note: The terms weight-for-length Z score (WLZ) and weight-for-height Z score (WHZ) are interchangeable.

Source: WHO and UNICEF (2009). WHO child growth standards and the identification of severe acute malnutrition in infants and children: A joint statement. Geneva/New York: WHO/UNICEF.

Adapted from Emergency Nutrition Network: The Current State of Evidence and Thinking on Wasting Prevention. 2018.

**Supplemental Table 3**. COREQ Criteria (uploaded as a PDF).

**Supplemental Table 4**. Codebook for key informant interview analysis

| Code Name | | Meaning |
| --- | --- | --- |
|  | |  |
| Intervention Types | | The different intervention examples mentioned by respondents |
| Nutrition Education | | Specific interventions that focus on nutrition education |
| Nutrition Supplementation | | Specific interventions that focus on provision of food or food supplements |
| Categorization of Interventions | | How interventions are categorized by respondent |
| Nutritional Status | | References to how nutritional status affected targeting of an intervention |
| Child age | | References to how age affected targeting of an intervention |
| Multiple outcomes | | Description from respondent about how an intervention affects multiple nutrition outcomes |
| Wasting and Acute | | Terms and definitions used to describe acute malnutrition compared to wasting |
| Importance MAM | | Description of the relative importance that organizations place on wasting prevention versus other nutrition problems, including reasons |
| Wasting prevention recognition | | Does respondent think the term is or would be recognized |
| Wasting prevention examples | | Examples of interventions that address wasting prevention |
| Camps | Descriptions of any division between “camps”, and “players” in the nutrition community with regards to wasting, stunting, prevention, treatment, etc. Includes reasons for these divisions. |  |
| Harmonization | Ideas for building harmonization between camps/groups |  |

1. StatCompiler is available at: https://dhsprogram.com/data/STATcompiler.cfm [↑](#footnote-ref-1)
2. https://www.humanitarianresponse.info/fr/node/144295 [↑](#footnote-ref-2)
3. http://rchiips.org/nfhs/pdf/NFHS4/India.pdf [↑](#footnote-ref-3)
